# Rituximab Not Effective for Hearing Loss in Cogan's Syndrome

**DOI:** 10.1155/2016/8352893

**Published:** 2016-10-24

**Authors:** Daniel R. Bunker, Leslie Dubin Kerr

**Affiliations:** Department of Medicine, Division of Rheumatology, Icahn School of Medicine at Mount Sinai, New York, NY 10029, USA

## Abstract

*Importance*. Rituximab was not effective in ameliorating the hearing loss in a patient with atypical Cogan's syndrome.* Observations*. We report the case of a patient who developed acute bilateral uveitis and sensorineural hearing loss. A diagnosis of atypical Cogan's syndrome was made. The patient's hearing loss did not improve despite high dose steroids and azathioprine. Rituximab was administered given a recent report of its efficacy in a patient with refractory disease; however, our patient's hearing loss did not improve.* Conclusion*. Hearing loss in Cogan's syndrome is difficult to treat. Though rituximab was ineffective in our case, earlier administration in the disease course could be effective for future patients.

## 1. Introduction

Cogan's syndrome is a rare disease characterized by inflammatory eye disease and vestibuloauditory dysfunction. The ocular symptoms are usually amenable to treatment but the vestibuloauditory symptoms can persist despite aggressive therapy. Rituximab has been proposed as a treatment option for persistent hearing loss associated with Cogan's syndrome; however, we report a case in which a patient's sensorineural hearing loss was not responsive to this agent.

## 2. Report of Case

A 43-year-old female was transferred to our institution because of bilateral hearing loss associated with bilateral eye pain and blurry vision. She had been in her usual state of health until five days earlier when she developed fevers, diarrhea and vomiting, dizziness, and diffuse myalgia/arthralgia. She presented to an outside hospital and was found to be tachycardic to 110 beats/minute with acute kidney injury (creatinine 2.1 mg/dL, previously normal) and thrombocytopenia (32 × 10^3^/*μ*L). She was treated with IV hydration, ciprofloxacin, and metronidazole, with improvement in her clinical and laboratory parameters. However, on hospital day 2, she developed acute onset bilateral hearing loss, associated with low grade tinnitus. She denied otalgia, otorrhea, vertigo, or ataxia. Concurrently, she endorsed bilateral eye redness, pain with eye movement, and decreased vision.

On arrival to our institution, she denied chronic fevers, night sweats, or weight loss; there was no history of chronic sinusitis or oral or genital ulcers, there was no history of chronic skin rashes or photosensitivity, there was no chronic arthralgia or arthritis, and there was no history of peripheral numbness/tingling or focal muscle weakness. She had a history of migraine headaches but was on no chronic medications. There was no family history of autoimmune disease. The patient was originally from Jamaica but had lived in the United States for 30 years. She worked in tax preparation without any known occupational exposures.

**Figure 1:**
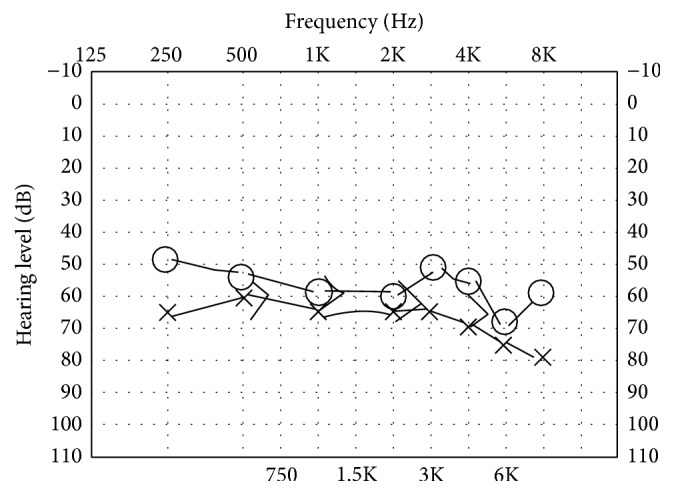
Audiogram at diagnosis.

On exam, she was afebrile with a normal heart rate and blood pressure in no acute distress. There were no facial rashes and her neurologic exam including cranial nerves was intact. Audiometry demonstrated bilateral sensorineural hearing loss (see Figure 1) and ophthalmologic exam showed bilateral anterior uveitis. Labs were notable for elevated inflammatory markers; however, specific connective tissue disease serologies (ANA, rheumatoid factor, ANCA, anticardiolipin, beta 2 glycoprotein, lupus anticoagulant, complements, and cryoglobulins) were normal. Rapid-plasma reagent, HIV, toxoplasma IgM, Lyme serologies, and CMV PCR were negative, but* Chlamydia trachomatis* IgG was 1 : 512 (*C. pneumoniae* and* C. psittaci* were negative) and* Mycoplasma* IgM was positive. MRI/MRA of the brain was unremarkable. CT angiography of the chest showed no vascular or pulmonary abnormalities.

**Figure 2:**
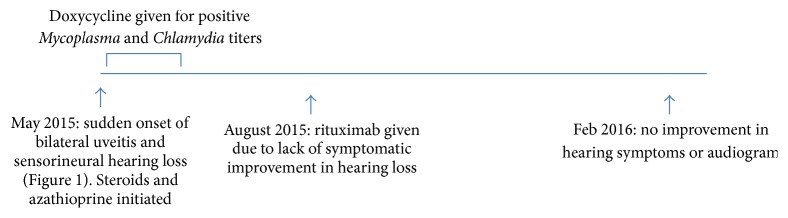
Patient's clinical course.

Given the inflammatory eye disease and bilateral sensorineural hearing loss and lack of signs of other autoimmune diseases, the patient was diagnosed with atypical Cogan's syndrome. She was treated with methylprednisolone 1 gm daily IV × 3 days and subsequently started on prednisone 1 mg/kg daily with azathioprine titrated to 150 mg daily as a steroid sparing agent. She was also treated with one month of doxycycline for her positive* Chlamydia* and* Mycoplasma* titers. Her hearing loss stabilized but did not improve after 3 months. Given the suggestion of efficacy of rituximab in sensorineural hearing loss [1], a report of improvement with this agent in a previous patient with Cogan's syndrome who had not responded to multiple other medications [2], and recent histologic findings of small vessel vasculitis in the pathology of this disease (for which rituximab is a well-established treatment) [3], rituximab was given at a dose of 375 mg/m^2^ weekly × 4 doses. At 6-month follow-up, however, her hearing had not improved and audiogram was unchanged. She is currently clinically stable and functions well with hearing aids.  Figure 2 depicts her clinical course.

## 3. Discussion

In 1945, ophthalmologist David Cogan reported a series of four patients with nonsyphilitic interstitial keratitis and audiovestibular dysfunction [4]. An association with a large vessel vasculitis resembling Takayasu's arteritis in up to 20% of patients was soon recognized. In 1980, the term “atypical Cogan's disease” was coined to recognize the patients with other types of inflammatory eye disease, including uveitis, as well as patients with a prolonged period between the development of ocular manifestations and that of vestibular manifestations [5]. Up to half of patient's with Cogan's syndrome do not have interstitial keratitis [5], as in our patient. This disease is rare and in the last 15 years there have only been two large series published, one from France in 2004 with 32 patients [6] and another from Mayo Clinic in 2006 with 60 patients [7]. The diagnosis is made when inflammatory ocular disease is found in patients with sensorineural hearing loss, and other potential etiologies are excluded.

The etiology of Cogan's syndrome is unknown. A large percentage of patients seem to have a preceding infectious illness, as in our patient.* Chlamydia* in particular has been proposed to be a triggering agent based on its ability to cause ocular infections as well as serologic evidence of recent* Chlamydia* infections in a high number of patients in previous series of Cogan's syndrome [8]. Our patient had high titer* Chlamydia* IgG, so we elected to treat our patient with antibiotics, based on data from reactive arthritis, which suggests clinical benefit if the underlying inflammatory stimulus is treated [9]. Interestingly, our patient was also found to have a positive* Mycoplasma* IgM antibody titer. While* Mycoplasma* has not been specifically identified as a precipitating factor in Cogan's syndrome, associations have been reported both with anterior uveitis [10] and with acute sensorineural hearing loss [11].

The pathophysiology of the vestibular symptoms in Cogan's syndrome has been unclear. Some studies have found an association with autoantibodies. In 2002, Lunardi et al. [12] found IgG antibodies in the serum of patients with Cogan's syndrome that bound antigens important in cell-cell communication in the inner ear; passive transfer of these antibodies to mice induced audiovestibular dysfunction. Additionally, antibodies to Heat Shock Protein- (HSP-) 70 have been detected in patients with sensorineural hearing loss, and a recent report found the presence of the antibodies to be highly associated with typical Cogan's syndrome [13]. However, in the largest series to date, only 1/10 patients tested positive for anti-HSP-70 antibodies [7]. Histopathologic analysis has been hampered by the inaccessibility of the inner ear in live patients. Autopsy studies in patients with Cogan's syndrome have found neuronal atrophy and degradation, bony proliferation, and inner ear atrophy [14], though these studies were generally conducted years after the initial symptoms (and after years of treatment), making the ability to draw conclusions difficult. A notable case report was recently published of a patient with Cogan's syndrome who expired less than three weeks after developing bilateral sensorineural hearing loss. Autopsy of the cochlea and vestibular system showed lymphocytic infiltration of vessel walls as well as vessel necrosis in both the cochlea and vestibular vessels [3], consistent with a small vessel vasculitis.

Though the ocular manifestations of Cogan's syndrome usually respond to topical therapies or systemic corticosteroids, the treatment of vestibuloauditory manifestations can be challenging. Up to half of patients become permanently deaf despite treatment [7], and prolonged vestibular abnormalities are common. Corticosteroids are the mainstay of treatment for sensorineural hearing loss and may be effective if they are administered early [15], but there is little consensus in the literature about the utility of additional immunosuppressive agents for these symptoms. Azathioprine, methotrexate, cyclosporine, and cyclophosphamide have been used with varying success; treatment with infliximab in refractory cases has shown promising results [16]. In 2010, Orsoni et al. described the case of a 25-year-old Italian woman with interstitial keratitis and bilateral sensorineural hearing loss whose hearing did not improve despite treatment with corticosteroids, cyclophosphamide, methotrexate, cyclosporine, and adalimumab. Rituximab 500 mg IV every 4 weeks was given and the patient experienced significant hearing improvement after 1 month [2]. Unfortunately, our patient's hearing did not improve with this agent, though it has stabilized. It is possible that rituximab was administered too late in the disease course, after the vasculitic inflammation had already caused irreversible damage to the vulnerable inner ear structures. More research is needed into the pathogenesis of Cogan's syndrome and the appropriate therapeutic strategy.
